# Supplementary figures and images for: A small molecule that mitigates bacterial infection disrupts Gram-negative cell membranes and is inhibited by cholesterol and neutral lipids
Source: PLoS Pathog. 2020 Dec 8;16(12):e1009119. doi: 10.1371/journal.ppat.1009119 (PMC7748285; doi:10.1371/journal.ppat.1009119)

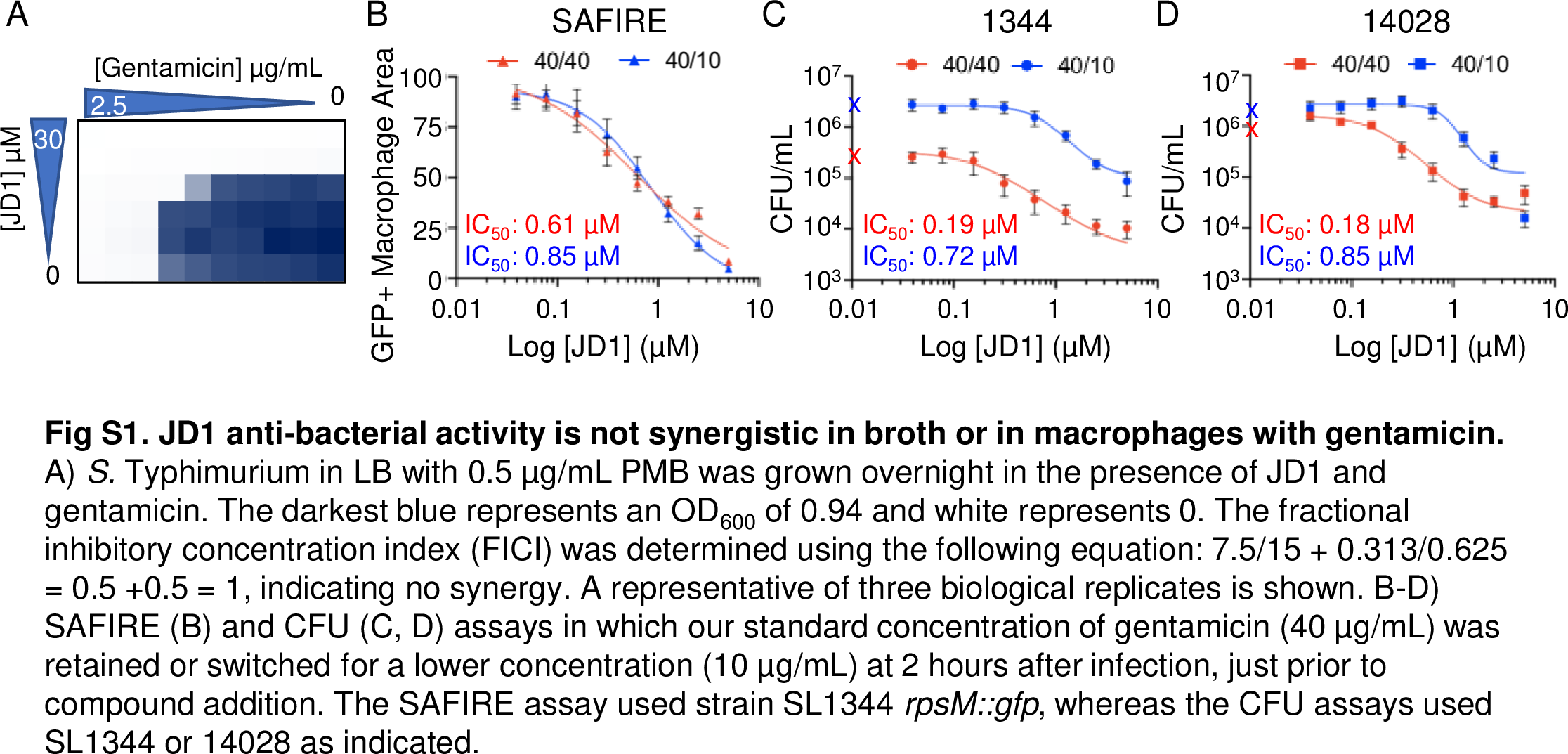

Supplement: S1 Fig — A) S. Typhimurium in LB with 0.5 μg/mL PMB was grown overnight in the presence of JD1 and gentamicin. The darkest blue represents an OD600 of 0.94 and white represents 0. The fractional inhibitory concentration index (FICI) was determined using the following equation: 7.5/15 + 0.313/0.625 = 0.5 +0.5 = 1, indicating no synergy. A representative of three biological replicates is shown. B-D) SAFIRE (B) and CFU (C, D) assays in which our standard concentration of gentamicin (40 μg/mL) was retained or switched for a lower concentration (10 μg/mL) at 2 hours after infection, just prior to compound addition. The SAFIRE assay used strain SL1344 rpsM::gfp, whereas the CFU assays used SL1344 or 14028 as indicated. (TIF) [file ppat.1009119.s001.tif]

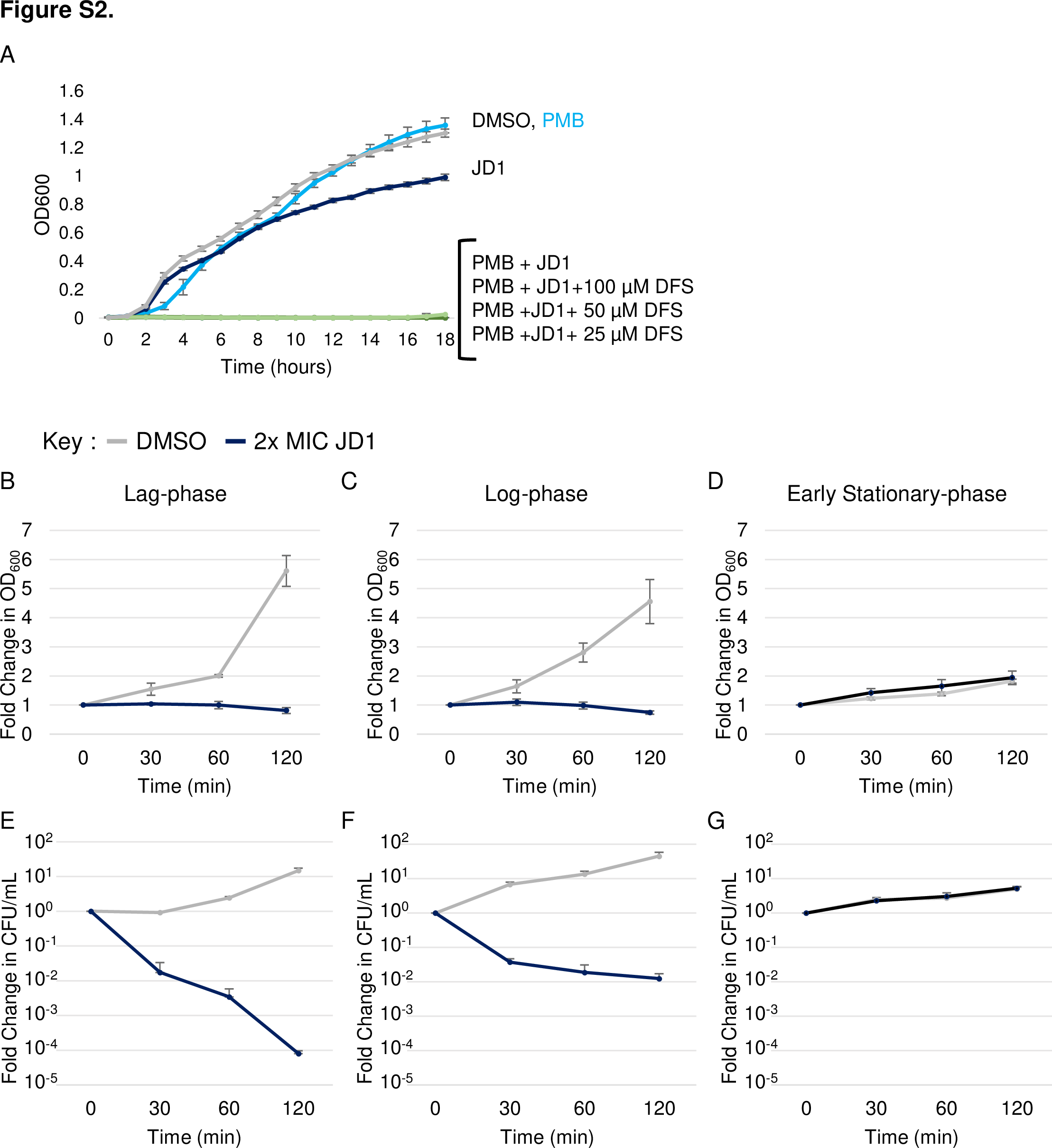

Supplement: S2 Fig — A) Treatment with the iron chelator deferasirox (DFS) does not rescue growth inhibition by JD1 (1x MIC) in LB with 0.5 μg/mL PMB. Mean and SEM of four biological replicates performed with technical triplicates. B-E) JD1 inhibits growth and kills cells in log-phase and lag-phase but not in early stationary phase. Overnight cultures were diluted 1:100 into LB with 0.5 μg/mL PMB and grown to the following ODs prior to the addition of DMSO or JD1 at 2x MIC (28 μM): ~0.1 (lag phase; B, E), 0.4–0.5 (log phase; C, F), or 1.0–1.4 (early stationary phase; D, G). (B-D) Cultures were monitored for OD600. (E-G) Cultures were plated for enumeration of CFU. Mean and SEM of three biological replicates. (TIF) [file ppat.1009119.s002.tif]
