# Supplementary material for: A small molecule that mitigates bacterial infection disrupts Gram-negative cell membranes and is inhibited by cholesterol and neutral lipids
Source: PLoS Pathog. 2020 Dec 8;16(12):e1009119. doi: 10.1371/journal.ppat.1009119 (PMC7748285; doi:10.1371/journal.ppat.1009119)
Supplement: Attachment — Submitted filename: Response_Sep30b_2020.docx [file ppat.1009119.s018.docx]

**Response to reviewers:**

We appreciate the thoughtful reviewer comments that have led to the following major changes and clearly improved the manuscript:

1) New experiments:

a) Fig 1 D. Demonstration that *S.* Typhimurium strain 14028, in addition to strain SL1344, is sensitive to JD1 in RAW264.7 cells on the basis of plating for CFU.

b) Fig 1 E, F. Demonstration in HeLa cells that *S.* Typhimurium is sensitive to JD1 growth inhibition on the basis of SAFIRE (SL1344 with the *rpsM::gfp* reporter) and plating for CFU (SL1344 and 14028).

c) Fig S1 A. Checkerboard indicating a lack of synergy between JD1 and gentamicin in broth.

d) Fig S1 B. SAFIRE assays in RAW264.7 cells showing that reducing the concentration of gentamicin during infection does not change the JD1 dose response curve.

e) Fig S1 C, D. CFU assays in RAW264.7 cells with bacterial strains SL1344 and 14028 demonstrating that the CFU curves shift up with less gentamicin in a JD1-independent manner, consistent with the use of gentamicin to reduce the number of extracellular bacteria and inconsistent of gentamicin-JD1 synergy.

f) Fig S2A. Graph showing that an iron chelator does not diminish the impact of JD1 on bacterial growth in broth.

g) Fig S2 B-G. Graphs illustrating that JD1 affects lag- and log-phase cells similarly but not early stationary phase cells.

2) A re-working of the manuscript for clarity

3) An expansion of the discussion to more fully address the mechanism of action of JD1 in media versus macrophages

**Major Comments**

Reviewer #2: The MIC95 of JD1 for the Salmonella ∆acrAB mutant and the WT in different media compositions is approximately 100x that found within macrophages (as stated in Table 1). This finding suggests that there may be a significant dichotomy in the mechanism of JD1 in macrophages versus that found in media and may diminish the conclusions regarding membrane fluidity and efflux. A demonstration of the effect of efflux and membrane composition, either through chemical or genetic means, on the efficacy of JD1 against intracellular Salmonella would demonstrate relevance of mechanistic studies on inhibition of intracellular Salmonella. Furthermore, these discrepancies merit comment in the discussion.

We have modified the discussion to address the mechanism of action of JD1 in media versus macrophages, Line 478. It may be possible to use chemical or genetic means in macrophages to tease out the relative contributions of JD1 activity on membranes versus efflux pumps. Since these experiments will be quite involved due to the likely negative effects of chemicals and mutations on Salmonella replication in macrophages, they are appropriate for a future manuscript.

Correlation of the in vitro derived mutants with improved compound resistance in macrophages would strengthen the conclusions of the manuscript regarding efflux.

The resistant mutants were not fit with regard to replication in macrophages, with GFP+ signal ranging from 4 – 61% of WT (see table below). This may reflect that efflux pumps are required for *S.* Typhimurium survival in macrophages and dysregulation may reduce bacterial replication. Alternatively, or in addition, other mutations that accumulated during selection may play a role. Some of the mutants may be resistant to JD1; mutant 2 had only ~5-fold fewer GFP+ bacterial compared to ~50-fold for wild-type at 4 µM JD1. Definitive conclusions will require genetic separation of fitness and resistance effects, if possible, and could go in a follow-up report.

| **Treatment** | **% WT DMSO GFP+ Macrophage Area** | | | | | | |
| --- | --- | --- | --- | --- | --- | --- | --- |
|  | **WT** | **M1** | **M2** | **M3** | **M4** | **M5** | **M6** |
| **DMSO** | 100 ± 1.3 | 3.5 ± 0.7 | 22.4 ± 0.8 | 18.4 ± 3.2 | 61.0 ± 3.6 | 46.2 ± 7.0 | 13.7 ± 0.9 |
| **2 µM JD1** | 6.7 ± 1.4 | 2.5 ± 0.2 | 9.6 ± 0.4 | 5.5 ± 0.1 | 5.5 ± 0.3 | 3.8 ± 0.6 | 4.8 ± 0.0 |
| **4 µM JD1** | 2.2 ± 0.5 | 0.3 ± 0.2 | 4.7 ± 0.5 | 1.9 ± 0.7 | 1.4 ± 1.0 | 1.0 ± 0.5 | 2.5 ± 0.4 |

Together the data demonstrate an effect of JD1 on membranes of Gram-negative bacteria, mitochondria, and eukaryotic cells. While the concentrations needed to cause gross changes to macrophages are higher than those needed to reduce intracellular Salmonella, it is unclear whether minor membrane damage caused by JD1 could increase permeability of macrophages to gentamicin in the assay buffer and therefore the efficacy of JD1 could represent sensitivity of intracellular Salmonella to gentamicin rather than to JD1. Experiments to rule out this potential confounding factor would strengthen the conclusions of the manuscript regarding the efficacy of JD1 against intracellular Salmonella.

We performed checkerboard broth growth experiments and found that JD1 and gentamicin do not inhibit growth synergistically (Fig S1 A). To address in-macrophage synergy, we determined the potency of JD1 at two different concentrations of gentamicin in SAFIRE and CFU assays (Fig S1 B-D). The SAFIRE curves with low versus high gentamicin were similar. The SL1344 CFU samples with low gentamicin yielded more CFU than samples with high gentamicin independent of JD1. The 14028 samples with low gentamicin are similar to samples with high gentamicin at low and high concentrations of JD1. At concentrations of JD1 near the IC_50_, 14028 samples with high gentamicin have fewer CFU, suggesting the elimination of extracellular bacteria is most important in samples for which small differences in JD1 concentration yield large differences in bacteria intracellular survival.

Reviewer #3: 1) Although the intracellular activity of JD1 is of critical importance to the study, this was only demonstrated in RAW264.7 cells, a macrophage-like cell line that is quite permissive for intracellular replication of STm. What about in primary mouse macrophages? Or other relevant host cells such as epithelial cells. The role of antimicrobial peptides could be more thoroughly investigated using macrophages from mice deficient in the production of e.g. CRAMP or by the addition of purified peptides to the culture.

We now show that JD1 is potent against *S.* Typhimurium in a human HeLa epithelial cell line, in addition to a murine cell line (Fig 1 E, F); the SARS-CoV-2 fiasco has limited our access to animals. It is a good idea to investigate the role of endogenous antimicrobial peptides in potentiating JD1. This will include the quantitative knock down of RNA from CRAMP and possibly other host AMPs, the use of mouse lines deficient in host AMPs, and the addition of purified peptides to cultured macrophages. Given the complexity of these experiments and the possibility that CRAMP may not be the sole important AMP, they are suitable for a future manuscript.

2) The strain of STm used here, SL1344, is a histidine auxotroph that has impaired growth in macrophages (Henry et al Molecular Microbiology (2005) 56(1), 252–267). Does this contribute to the antimicrobial activity of JD1? Are other STm strains e.g. 14028 also sensitive to JD1? What happens if supplemental histidine, which relieves the growth defect of intracellular SL1344, is added to the culture media?

We obtain similar results with SL1344 and 14028 in RAW264.7 cells and in HeLas with SAFIRE and CFU assays (Fig 1D, F).

3) Is the effect of JD1 growth phase dependent? When testing the activity of JD1 against intracellular bacteria the drug was added at 2 h pi a time at which the bacteria are not actively replicating. The inoculum was prepared from overnight (stationary?) cultures and the bacteria do not replicate intracellularly until >4 h pi. In contrast, in the in vitro assays JD1 was primarily added during logarithmic growth. What is the effect of adding JD1 to cultured bacteria during lag phase rather than logarithmic?

We added a supplementary figure (Fig S2 B-G) showing that JD1 inhibits the growth of lag and log phase cells but not of early stationary phase cells.

**Minor Issues: Editorial and Data Presentation Modifications**

Reviewer #1: 1. Fig 1. You need to more clearly explain how you calculate GFP+ Macrophage Area. You describe how you acquire the data, but not how you get the underlying values which are then converted to a percent. Likewise for the Integrated Density.

Thank you, we have added explanations to the legend of Fig 1.

2. Table 1. Why is the IC95 given in two different units? Why is PMB listed ad “ug” and PMBN listed as “ug/ml”?

The PMB typo has been fixed. The values in Table 1 are provided in µM units as preferred by biochemists and in µg/mL as preferred by antibiotic developers. Whenever we use just one unit, at least one reviewer requests the other.

3. It is not clear that the isothermal calorimetry with purified AcrB adds much to the paper. Given that the protein is transmembrane, you must at least describe its purification and your confidence in it being in a physiological conformation. What is the binding affinity for some known substrate?

Thank you, we have put the ITC results into context in the results and provided the purification protocol in the methods.

4. Line 226 and 634. The methods sentence is awkwardly worded. It can be interpreted as saying that you took one single colony and inoculated 6 cultures. I assume that each independent culture was inoculated with a separate colony. Reword.

We apologize for the confusion and have re-worded the corresponding text for clarity.

5. Line 246. Did you ever test any of the efflux mutants in the macrophage assay? Not a big deal, just an interesting question.

Please see the comment and response regarding the mutants above.

6. Line 254. “With a LogP value of 12.5…” You need a reference here or some explanation of what LogP means. You explain it later, but not here.

Thank you, we now define LogP after its first use.

Reviewer #2: The MIC95 data presented in Table 1 are contradicted in the text and figures. Line 143 states MIC95 of 0.63 µM within macrophages whereas table 1 indicates a MIC95 of intracellular Salmonella of 1.2 µM by GFP fluorescence. Imaging of membrane morphology states usage of 1X MIC (line 312) and figure 6 shows 14 µM JD1 whereas the MIC95 listed in table 1 are 25.9 and 20.06 µM for each E. coli strain shown. Please clarify in methods or legends for figures 2, 4, and 5 the concentrations of JD1 used for each data set.

We have fixed incorrect values and clarified in the legends the concentrations of JD1 used.

Lines 239-40: “Upon removal of CCCP and the addition of glucose 5 of 6 mutant strains exported…” contradicts data presented in Table 2 that demonstrates increased efflux for 2 of 6 mutants upon addition of glucose. Please correct and comment upon potential mechanism for lack of response to glucose on efflux.

Thank you for noting the discrepancies. We have re-written this section for clarity and accuracy. We also normalized the +glucose column to the WT-glucose so that the response of the strains to the addition of glucose is apparent.

Lines 243-4: Are the resistant mutants 1-6 identified through in vitro acclimation resistant to the effects of JD1 in macrophages?

Please see the comment and response regarding the mutants above.

Line 353: Are clinical data available for mouse infections? Was there evidence of tissue damage in treated animals?

We did not observe tissue damage upon dissection. We did not collect clinical data for the mouse infections because JD1 is a hit and not a lead compound and the cost of pathological workups was prohibitive.

Lines 643-9: The sequencing results for mutants 1 and 4 should be described in the results.

We moved the text from the methods to the results.

Line 696: Please describe calculations used to determine intracellular pH.

Thank you, we added the equation to the methods.

Line 805-6: Thank you for indicating approval of compound administration dosages in animal use protocols. Please describe the rationale for JD1 dose and dosage regimen for mice. In what volume were drug and compound administered?

We have added the requested information to the methods.

Line 809 indicates collection of clinical data from mice. Please display these data in the manuscript.

We have corrected the error.

Line 1141: Please indicate the media in which the ∆acrAB mutant was evaluated.

We have clarified the media used in the figure legend.

Figure 4G: Y-axis describes data normalized to DMSO, but data suggest that DMSO increases above 1 with time. Please clarify.

Thank you, we have added “normalized to DMSO at time 0.”

Reviewer #3: 1) The manuscript would benefit from editing for brevity and clarity. The following are provided as examples:
- In the introduction, the paragraph about the SAFIRE screen is extraneous as it has already been published and could be reduced to a sentence citing the original paper.

Since the manuscript shows unpublished SAFIRE data (Fig 1, Fig S1), a description of the method is needed. This description is in the introduction and is not repeated in the results.
- The first two paragraphs of the intro are somewhat redundant.

Thank you, we have streamlined these two paragraphs.
- The lines 61-63 from the summary state, “This is the first compound, to our knowledge, that preferentially targets the cell membranes of Gram-negative bacteria and reduces bacterial infection of animals.” But lines 491-492 in the conclusion state “At least one lipophilic compound, metergoline, has a demonstrated ability to damage the cell membrane of Gram-negative bacteria and to inhibit infection.” These seem contradictory

Thank you, we have reworded the paragraph to more directly state that metergoline interacts with multiple eukaryotic cell surface receptors known to mitigate infection.
- Within the results section there are some inconsistencies that make it hard to follow or interpret the data. In particular, it is not always clear which membrane or membranes are being analyzed for each assay and ultimately which membrane JD1 is affecting. Also, the use of MIC is not consistent. Defined as the concentration at which 95% of growth was inhibited, lines 162-13, in other places they used phrases such as “completely unable to grow” (line 173) or “conc. Needed to inhibit growth” (line 186).

We searched on “membrane” and added “cell”, “inner”, “outer”, “phagosomal”, “host” or “mitochondrial” wherever it would not be redundant or incorrect. We also removed phrases referring to growth and replaced them with more precise statements regarding MIC.

- Several sections in the Methods are not fully clear. For example, in the evolution of resistance, how long were bacterial cultures grown? How was it determined if there was “visible growth”? OD600? Also, the text states that growth at 3X MIC took approximately 12 generations (line 637). Should this be passages rather than generations?

Thank you, we have made corrections throughout the methods accordingly.

2) The SifB::GFP reporter used in the SAFIRE screen has also been used here to demonstrate that JD1 blocks replication of intracellular bacteria. However, another inhibition of SPI2 induction within intracellular bacteria would also block GFP expression. Why not use a constitutive GFP reporter that would show the total intracellular population?

We use an *rpsM::gfp* reporter for the SAFIRE HeLa cell experiments (Fig 1E, Fig S1B) because the *sifB::gfp* reporter is not sufficiently expressed in these cells. However, this reporter increases the background of the SAFIRE experiments because extracellular bacteria that are GFP+ may be on top of cells (Reens 2018, Nagy 2019). We added a statement to the discussion that JD1 could in theory inhibit any process needed for bacterial replication or survival in cells (Line 483).

3) Several pieces of data seem to be missing from the manuscript.
- The iron chelator deferasirox

These data are now depicted in Fig S2A instead of stated in the text.

Hoechst staining

We now state in the text “mean +/- standard deviation”.

- Pictures or quantification of liver abscess

We did not collect clinical data for the mouse infections because JD1 is a hit and not a	lead compound and the cost of pathological workups was prohibitive.

4) There should be consistency and clarity in their statistics throughout the paper. Standard deviation should be used to convey the spread of variability of the data, not SEM, which tends to just show smaller error bars. Three biological replicates should be done for every figure. Some of the figures are lacking the statistical test used in the figure legend. Finally, Students t-test should not be used when more than two groups are being compare e.g. Fig 8

Thank you, we have clarified in the figure legends that SEMs are shown for data that are combined from n biological replicates, each performed with n technical replicates and that standard deviations are shown for representative experiments. Fig 8 compares only two data points at each time point, such that a t-test is appropriate.
